# Rhein Induces Cell Death in HepaRG Cells through Cell Cycle Arrest and Apoptotic Pathway

**DOI:** 10.3390/ijms19041060

**Published:** 2018-04-02

**Authors:** Longtai You, Xiaoxv Dong, Xingbin Yin, Chunjing Yang, Xin Leng, Wenping Wang, Jian Ni

**Affiliations:** 1 School of Chinese Materia Medica, Beijing University of Chinese Medicine, Beijing 100029, China; ylt_svip@163.com (L.Y.); dxiaoxv@163.com (X.D.); yxbtcm@163.com (X.Y.); 20160941191@bucm.edu.cn (C.Y.); 20160931927@bucm.edu.cn (X.L.); wangwenp6@163.com (W.W.); 2 Beijing Research Institute of Chinese Medicine, Beijing University of Chinese Medicine, Beijing 100029, China

**Keywords:** rhein, hepatotoxicity, HepaRG cells, ROS, apoptosis

## Abstract

Rhein, a naturally occurring active anthraquinone found abundantly in various medicinal and nutritional herbs, possesses a wide spectrum of pharmacological effects. Furthermore, previous studies have reported that rhein could induce hepatotoxicity in rats. However, its cytotoxicity and potential molecular mechanisms remain unclear. Therefore, the present study aimed to investigate the cytotoxicity of rhein on HepaRG cells and the underlying mechanisms of its cytotoxicity. Our results demonstrate, by 3-(4,5-dimethyl thiazol-2-yl-)-2,5-diphenyl tetrazolium bromide (MTT) and Annexin V-fluoresce isothiocyanate (FITC)/propidium iodide (PI) double-staining assays, that rhein significantly inhibited cell viability and induced apoptosis in HepaRG cells. Moreover, rhein treatm**e**nt resulted in the generation of reactive oxygen species (ROS), loss of mitochondrial membrane potential (MMP), and S phase cell cycle arrest. The results of Western blotting showed that rhein treatment resulted in a significant increase in the protein levels of Fas, p53, p21, Bax, cleaved caspases-3, -8, -9, and poly(ADP-ribose)polymerase (PARP). The protein expression of Bcl-2, cyclin A, and cyclin-dependent kinase 2 (CDK 2) was decreased. In conclusion, these results suggest that rhein treatment could inhibit cell viability of HepaRG cells and induce cell death through cell cycle arrest in the S phase and activation of Fas- and mitochondrial-mediated pathways of apoptosis. These findings emphasize the need to assess the risk of exposure for humans to rhein.

## 1. Introduction

**Figure 1:**
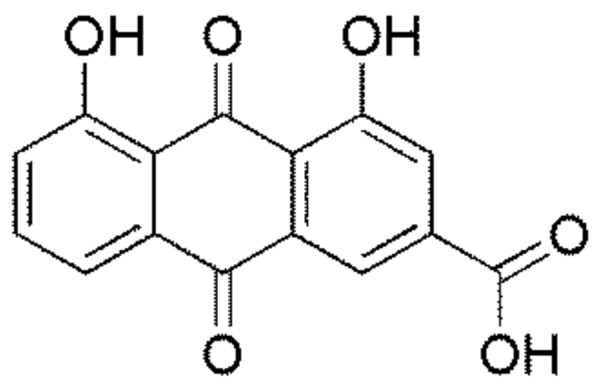
The chemical structure of rhein.

Rhein (4,5-dihydroxyanthraquinone-2-carboxylic acid) (see Figure 1) is an anthraquinone compound and is primarily separated from *Cassia occidentalis*, *Polygonum multiflorum*, and *Rheum palmatum* L., which have been widely used as a laxative or a stomachic agent in many countries for a long time [1,2]. Modern pharmacological studies have suggested that rhein possesses a number of biological properties including anticancer [3], antiviral [4], anti-inflammatory [5], and antimycobacterial effects [6]. Previous studies have shown that rhein inhibits the growth of various cells such as human tongue cancer cells (SCC-4), human lung cancer cells (A-549), human nasopharyngeal carcinoma cells (NPC), and human promyelocytic leukemia cells (HL-60) [2,7,8,9]. Furthermore, the expression of many proteins (PKR-like ER kinase (PERK), CCAAT/enhancer-binding protein homologous protein (CHOP0), Bcl-2, and caspase-3) that induce apoptosis have been shown to be regulated by rhein [10,11,12,13]. Some studies have demonstrated that rhein has cytotoxic effects in L-02 and HepG2 cells, which further reveal that rhein might be one of the major toxic ingredients [14,15]. Rhein has been reported to be involved in a series of mitochondrial functions including oxidative phosphorylation and inhibits oxidation of FAD- or NAD-linked substrates. Moreover, it mediates toxicity in rat primary hepatocytes through the generation of reactive oxygen species [16,17].

Apoptosis, which is a form of autonomic ordered programmed cell death, plays a critical role in maintaining homeostasis in normal human liver, which is regulated through a series of genes. It is genetically controlled by many correlative processes including the death receptor-mediated extrinsic pathway and the mitochondrial-dependent intrinsic pathway [18,19,20]. Caspases are a family of cysteine proteases that are well characterized as driving cell apoptosis or death [21]. The extrinsic pathway is initiated via ligation of the death receptors (Fas/Fas-L) and subsequent caspase-8 activation within a death-inducing signaling complex. In contrast, the intrinsic pathway is triggered by intracellular stress and is subsequently activated by the release of cytochrome c and caspase-9 activation. Even though the two pathways can be activated by diverse stimuli, both will directly trigger downstream effector caspase-3 and ultimately lead to cell apoptosis [22,23]. Moreover, the regulation and control of mitochondrial-dependent apoptotic events occur mainly through the Bcl-2 family proteins including Bcl-2, Bak, and Bax [24]. Caspases can be significantly activated by an increase in the Bax/Bcl-2 ratio, which then leads to programmed cell death through the mitochondrial-dependent apoptotic pathway [25].

The HepaRG cell line was derived from a female patient suffering from hepatitis C infection and hepatocellular carcinoma. The cell line is regarded as a superior surrogate in vitro model for assessing drug-induced hepatotoxicity since this cell line expresses high levels of various CYPs, such as detoxification enzymes (CYP3A4) and drug-metabolizing enzyme (CYP4F3B). It also possesses both the metabolic performance of primary human hepatocytes and the growth capacity of a hepatic cell line [26,27]. In this study, we elucidated the cytotoxicity of rhein in HepaRG cells in vitro. Our results suggest that rhein treatment could induce cell death through cell cycle arrest in the S phase and activation of Fas- and mitochondrial-mediated pathways of apoptosis.

## 2. Results

### 2.1. Rhein Induces Cytotoxicity in HepaRG Cells

**Figure 2:**
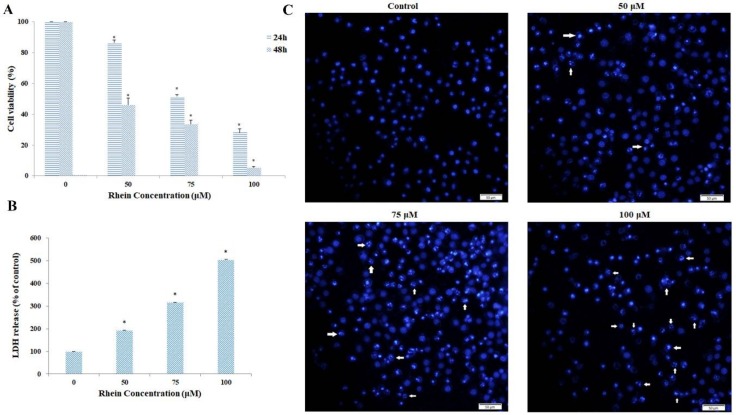
Effects of rhein on HepaRG cell viability. HepaRG cells were treated with rhein in a series of concentrations (0, 50, 75, 100 μM) for 24 h, 48 h, and 72 h. (**A**) Cell viability was assessed by the MTT assay. (**B**) Cell cytotoxicity was measured by the LDH assay. (**C**) The morphological changes in 24-h rhein-treated HepaRG cells following staining with fluorescent 4′,6-diamidino-2-phenylindole (DAPI) were observed by fluorescence microscopy (Original magnification = 200×, Bar = 50 µm). Arrows indicate bright blue apoptotic cells. Results are the mean ± S.D. (*n* = 3). LSD *t*-test was carried out. * *p* < 0.05, significantly different compared with vehicle control.

Compared with the vehicle controls, the results of the 3-(4,5-dimethyl thiazol-2-yl-)-2,5-diphenyl tetrazolium bromide (MTT) assay demonstrated that rhein remarkably inhibited cell viability in a dose-dependent and time-dependent manner (see Figure 2A). The IC_50_ value of rhein for 24 h was 77.97 μM for HepaRG cells. Lactate dehydrogenase (LDH) is present mainly in the cytoplasm and exists in the extracellular medium, which is used to investigate damage in cell membrane integrity. LDH leakage is considered as a sign of cell membrane disruption. The experimental results show that rhein treatment resulted in a dose-dependent increase in LDH leakage from HepaRG cells (see Figure 2B).

### 2.2. Rhein Induces Apoptosis in HepaRG Cells

**Figure 3:**
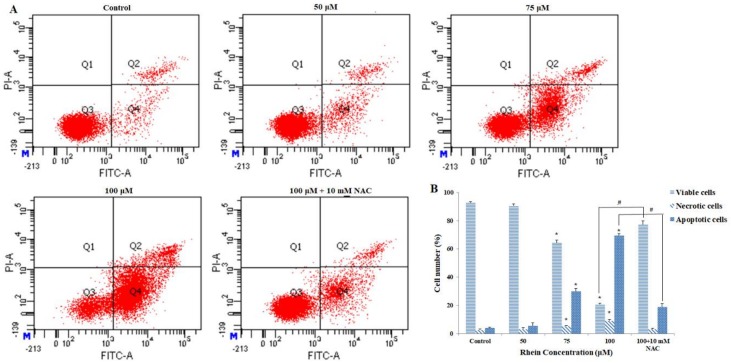
HepaRG cells were treated with various concentrations of rhein (0, 50, 75, 100 μM) for 24 h. (**A**) Annexin V-FITC/PI double staining was performed using a flow cytometer. (**B**) Column bar graph of the mean cell fluorescence for viable, apoptotic, and necrotic cells. The total percentage of apoptotic cells is expressed as the summation of both early and late apoptosis subpopulations. Results are the mean ± S.D. (*n* = 3). LSD *t*-test was carried out. * *p* < 0.05, significantly different compared with vehicle control. ^#^ *p* < 0.05, significantly different compared with rhein 100 μM-treated group.

To determine whether rhein inhibited the growth of HepaRG cells by inducing apoptosis, we performed DAPI staining and flow cytometry assays. Figure 2C shows rhein-induced apoptotic nuclear fragmentation and condensation of chromatin, which is clearly observed by DAPI staining. Annexin V-FITC/PI double staining was used to quantify the apoptotic cells. Compared with the control group, the proportion of viable cells was significantly lower after exposure to different concentrations of rhein for 24 h. Meanwhile, the proportion of apoptotic cells (early and late) and necrotic cells significantly increased in a dose-dependent manner (Figure 3A,B). In addition, when 50, 75, and 100 μM rhein was used, the *p*-values between the percentage of apoptosis and necrosis were 0.095, <0.000, and <0.000, respectively. Overall, these results clearly suggest that rhein can inhibit the growth of HepaRG cells by inducing apoptosis.

### 2.3. Effects of Rhein on Intracellular ROS and GSH Levels

**Figure 4:**
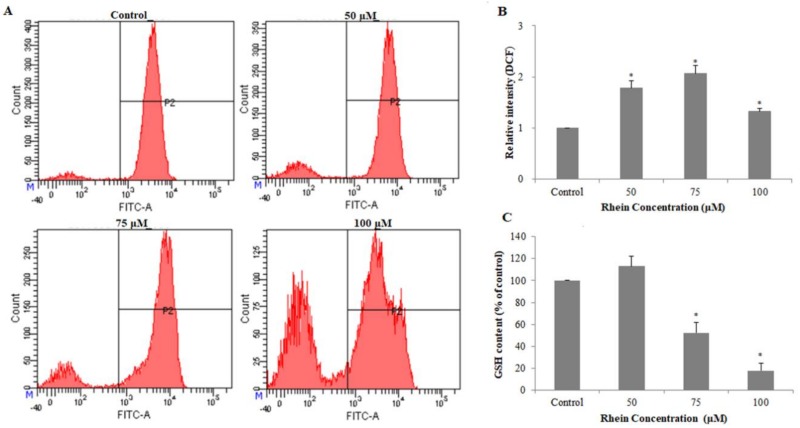
The levels of ROS and GSH in HepaRG cells were induced by different concentrations of rhein for 24 h. (**A**) The proportion of cells with ROS were stained with 2,7-dichlorofluorescin diacetate (DCFH-DA) dye and measured using a flow cytometer. (**B**) Histogram of average cell fluorescence of DCFH-DA. (**C**) The proportion of GSH in HepaRG cells after treatment with various concentrations of rhein for 24 h. Results are the mean ± S.D. (*n* = 3). LSD *t*-test was carried out. * *p* < 0.05, significantly different compared with vehicle control.

Previous studies have shown that reactive oxygen species (ROS), generated mainly by the mitochondria, can induce oxidative stress to regulate apoptosis, which consists of cell death [28,29]. To investigate whether rhein induces oxidative stress, the effects of rhein on intracellular ROS and reduced glutathione (GSH) levels were measured. As shown in Figure 4A,B, ROS generation was significantly increased by treating with rhein at a series of concentrations for 24 h. GSH plays an important role in many critical cellular processes including neutralizing ROS and maintaining the redox status. Therefore, changes in GSH levels can be monitored as a marker of cellular oxidative stress [30]. Compared with control, rhein treatment led to a significant decrease in GSH levels in HepaRG cells in a dose-dependent manner (see Figure 4C). Furthermore, pretreatment with the ROS inhibitor *N*-acetylcysteine (NAC) (10 mM) effectively blocked rhein-induced apoptosis in HepaRG cells (see Figure 3A,B). These results reveal that alterations in the levels of ROS and GSH may be involved in rhein-induced apoptosis by interfering with the cellular redox status.

### 2.4. Rhein Decreases MMP in HepaRG Cells

**Figure 5:**
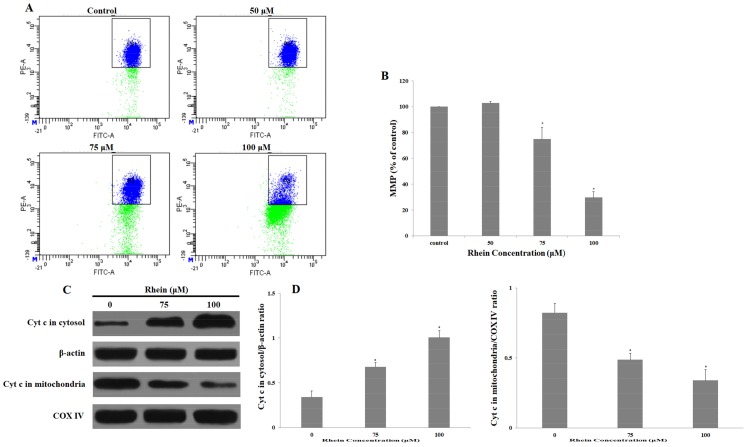
Depolarization of the mitochondrial membrane in HepaRG cells induced by different concentrations of rhein for 24 h. (**A**) The proportion of cells with MMP were stained by 5,5′,6,6′-tetrachloro-1,1′,3,3′-tetraethylbenzimidazolylcarbocyanine iodide (JC-1) dye and measured by flow cytometry; (**B**) Histogram of the average cell fluorescence of JC-1; (**C**) Expression of cytochrome c in the mitochondria and cytosol were determined by Western blotting. β-actin and COX IV expression levels served as an internal control in the cytoplasmic and mitochondrial fractions, respectively; (**D**) Quantity One software was used to quantify these protein-related bands. Results are the mean ± S.D. (*n* = 3). LSD *t*-test was carried out. * *p* < 0.05, significantly different compared with vehicle control.

In mitochondria-mediated apoptosis, the loss of mitochondrial membrane potential (MMP) leads to an increase in mitochondrial outer membrane permeability (MMOP), which then leads to mitochondrial dysfunction and the release of cytochrome c from the mitochondria to the cytoplasm [31,32]. Compared with the control group, the loss of MMP was clearly induced by rhein in a dose-dependent manner after incubation with various concentrations of rhein for 24 h (see Figure 5A,B). Moreover, this study further investigated whether incubating with rhein would strengthen the release of cytochrome c into the cytoplasm. The results indicate that the release of cytochrome c into the cytosol markedly increased while cytochrome c significantly decreased in the mitochondria after 24 h of treatment with rhein (see Figure 5C,D). In general, these studies demonstrate that mitochondrial dysfunction likely participated in the rhein-induced apoptosis of HepaRG cells.

### 2.5. Rhein Elicits DNA Fragmentation and S Phase Cell Cycle Arrest in HepaRG Cells

**Figure 6:**
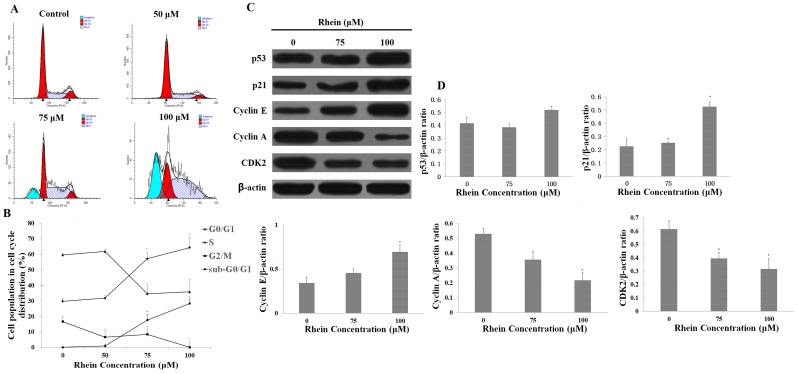
Flow cytometry was used to detect the effect of rhein on the cell cycle distribution of HepaRG cells. (**A**) Rhein induced S phase arrest after treatment with various concentrations of rhein for 24 h; (**B**) Each phase of the cell cycle is displayed in the histogram; (**C**) The expression levels of cell cycle-regulated proteins were determined by Western blotting. The β-actin was used as a loading control; (**D**) Quantity One software was used to quantify these protein-related bands. Results are the mean ± S.D. (*n* = 3). LSD *t*-test was carried out. * *p* < 0.05, significantly different compared with vehicle control.

To investigate whether rhein inhibits the proliferation of HepaRG cells by triggering cell cycle arrest, we determined the cell cycle distribution of rhein-treated cells by using flow cytometry. Moreover, by detecting the presence of the sub-G0/G1 cell population, the DNA fragmentation that occurs during the late stages of apoptotic cell death could be observed. From Figure 2, which demonstrated rhein-inducing apoptosis, we proceeded to detect the presence of the sub-G0/G1 cell population corresponding to apoptotic DNA fragmentation (see Figure 6A,B). Meanwhile, rhein resulted in an increase in cells in the S phase and a corresponding decrease in G0/G1 and G2/M phases in HepaRG cells when compared with untreated cells. To further explain the mechanisms, we examined the expression levels of the correlative proteins involved in the cell cycle progression. After 24 h of rhein (100 μM) exposure, rhein markedly upregulated the expression levels of cyclin E, p53, and p21 proteins and downregulated cyclin A and CDK2 proteins (see Figure 6C,D). These results indicate that rhein can induce S phase arrest in HepaRG cells by altering the key molecular markers of S cell cycle regulation and show apoptotic DNA fragmentation.

### 2.6. Effects of Rhein on Levels of Apoptosis-Regulated Proteins in HepaRG Cells

**Figure 7:**
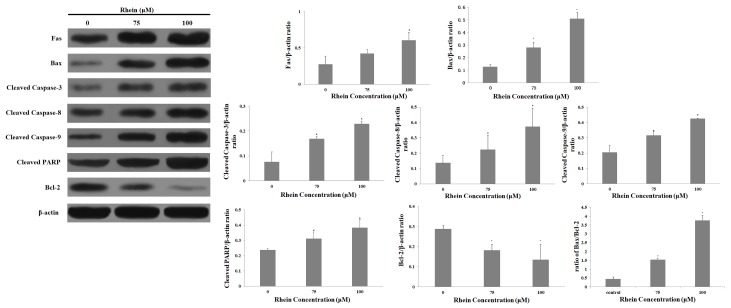
Western blotting was used to detect the expression of apoptosis-related proteins in HepaRG cells after treatment with various concentrations of rhein for 24 h. The β-actin was used as a loading control. Quantity One software was used to quantify the protein-related bands. Results are the mean ± S.D. (*n* = 3). LSD *t*-test was carried out. * *p* < 0.05, significantly different compared with vehicle control.

Apoptosis can be stimulated and triggered either via the cell surface death receptor-mediated extrinsic pathway or the mitochondrial-dependent intrinsic pathway [18,19,20]. Therefore, we attempted to explore the pathway of rhein-induced apoptosis and to further elucidate the potential mechanism. The expression of apoptosis-regulated proteins was measured by Western blot. As shown in Figure 7, rhein significantly increased the protein expression of Bax, cleaved caspase-3, -8, and -9, and resulted in the cleavage of poly(ADP-ribose)polymerase (PARP), which is the known substrate of caspase-3. Meanwhile, Bcl-2 is an anti-apoptotic protein that was clearly downregulated by 24 h treatment with rhein. In addition, we also investigated Fas protein, which is one of the representative members of the extrinsic pathway. Figure 7 shows that rhein increased the expression of Fas. These results suggest that rhein activated the intrinsic and extrinsic pathways of apoptosis.

## 3. Discussion

Modern pharmacological studies have shown that rhein possesses many significant therapeutic effects such as anti-inflammatory, antioxidant, antitumor, and antifibrosis [1,2,3,4,5,6]. However, previous studies have suggested that rhein might be one of the major hepatotoxic and nephrotoxic ingredients in *Rheum palmatum* L. [33]. In this study, we used the HepaRG cell line to investigate the cytotoxicity of rhein and its potential molecular mechanisms.

Compared with the control group, the results of MTT and LDH demonstrated that rhein inhibited HepaRG cell viability in a dose-dependent and time-dependent manner. Moreover, nuclear condensation and cell shrinkage were observed by DAPI staining. Annexin V/PI double staining further demonstrated that rhein treatment dose-dependently increased the number of apoptotic cells by inducing apoptosis. These results indicate that rhein can induce apoptosis in HepaRG cells.

A minor increase in the level of intracellular ROS can activate cell proliferation while excessive ROS generation leads to lipid peroxidation and structural changes of relevant proteins to further cause cell death, which is a key event in apoptosis [34,35]. Intracellular GSH is a major non-protein antioxidant that maintains the detoxification system by eliminating excessive ROS and recycling antioxidants. Our studies have shown that excessive ROS can participate in rhein-induced apoptosis. It was speculated that rhein treatment interferes with cellular redox status. In previous studies, ROS-induced apoptosis modulated the ratio of Bax/Bcl-2 expression, which stimulated mitochondrial membrane depolarization and the release of cytochrome C from the mitochondria into the cytosol [36]. In the present study, rhein treatment significantly stimulated the generation of ROS and decreased the level of GSH, which indicates that oxidative stress existed in the HepaRG cells. Moreover, rhein induced the loss of MMP in HepaRG cells, increased the Bax/Bcl-2 protein ratio, and promoted the release of cytochrome c from the mitochondria into the cytosol. Therefore, we conclude that ROS plays an important role in mitochondrial damage caused by rhein.

It is widely believed that there is a close relationship between oxidative stress and DNA damage [37]. The production of excess ROS can induce oxidative damage to DNA, such as nucleotide modifications and strand breaks especially in high guanosine content [38]. When severe DNA damage and oxidative damage are encountered, p53 can be rapidly activated and can accumulate in large amounts in the nucleus [39]. Furthermore, previous studies have shown that p53 can induce apoptosis by regulating apoptosis-related proteins including Bax and Bcl-2 [40]. Our study shows that rhein treatment can increase the expression of p53 and the ratio of Bax/Bcl-2 expression. These results indicate that the excessive production of intracellular ROS could induce DNA damage, which indirectly leads to activation of the mitochondrial apoptotic pathway.

CDK2, which binds to the cyclins such as cyclin E, is responsible for chromosomal duplication. Previous studies showed that rhein inhibited the proliferation of cells (A-549 and SCC-4 cell lines) through cell cycle arrest [9,41]. Rhein increased the levels of p53 and p21 in A-549 cells and possessed an anti-cancer effect on SCC-4 cells by down-regulating cyclin A, which is consistent with the findings in the present study. Our data suggest that rhein induced the inhibition of HepaRG cell growth through S phase cell cycle arrest.

Apoptosis can be controlled through the death receptor-mediated pathway (extrinsic) and the mitochondrial-mediated pathway (intrinsic) [42,43]. However, both the extrinsic and intrinsic pathways activate effector caspases such as caspase-3 and caspase-8, which ultimately leads to apoptosis. Previous studies suggested that rhein enhanced the expression of Fas/death-receptor in human cervical cancer Ca Ski cells through the extrinsic pathway and increased the ratio of Bax/Bcl-2 in human aortic smooth muscle cells through the intrinsic pathway [44,45]. These results are all in accord with the findings in our study, which suggest that rhein treatment resulted in a significant increase in Fas, Bax, and cleaved caspase-3, -8, and -9 and a downregulation in the expression level of Bcl-2 when compared with the untreated cells. PARP is a major downstream substrate of caspase-3, which directly leads to apoptosis [46]. Our study shows a significant increase in PARP cleavage after treatment with various doses of rhein. Currently, our results show that rhein-induced apoptosis in HepaRG cells involves the Fas death receptor-mediated and the caspase-dependent mitochondrial apoptotic pathways.

In conclusion, our investigation is the first to show that rhein can significantly inhibit the growth of HepaRG cells by triggering S phase arrest and results in cell death by inducing apoptosis mediated through the extrinsic and intrinsic apoptotic pathways. In previous studies, researchers paid more attention to the pharmacological activity and clinical application of rhein with little focus on its side effects and toxicity. We hope that our study can provide effective suggestions for the safety of rhein in clinical medicine.

## 4. Materials and Methods

### 4.1. Reagents and Antibodies

Rhein (batch No. 5045, purity >98.0%) was purchased from Shanghai Standard Biotech Co., Ltd. (Shanghai, China). Fetal bovine serum (FBS), 0.25% trypsin, penicillin, and streptomycin solution were obtained from Corning (Corning, NY, USA). RPMI 1640 medium, dimethyl sulfoxide (DMSO), PBS, and MTT were purchased from Solarbio (Beijing, China). The LDH Assay Kit, Annexin V-FITC Apoptosis Assay Kit, DAPI Assay Kit, MMP Assay Kit, ROS Assay Kit, and Cell Cycle Assay Kit were obtained from Beyotime (Nanjing, China). Reduced glutathione (GSH) was purchased from Nanjing Jiancheng Bioengineering Institute (Nanjing, China). Antibodies against Fas (#4233S), Bax (#5023T), Bcl-2 (#15071), p53 (#2524T), p21 (#2947T), cyclin A (#4656T), CDK 2 (#2546T), cleaved caspase-3 (#9661T), cleaved caspase-9 (#9501T), cytochrome c (#4280T), and PARP (#9542T) were purchased from Cell Signaling Technology (Beverly, MA, USA). Antibody against caspase-8 (#ab25901) was purchased from Abcam (Shanghai, China) (#ab25901).

### 4.2. Cell Cultures and Treatment

The HepaRG cell line was purchased from Shanghai Guan and Dao Biological Engineering Co., Ltd. (Shanghai, China). Cells were cultured in RPMI 1640 medium containing 10% (*v*/*v*) fetal bovine serum (FBS) and antibiotics (100 U/mL penicillin and 100 μg/mL streptomycin). The cells were maintained at 37 °C in a humidified atmosphere with 5% CO_2_. Trypsin (0.25%, Corning) was used to passage cells at 80–90% confluence. Rhein was dissolved in dimethyl sulfoxide (DMSO) to obtain a stock concentration of 20 mM and further dilutions were made with the basal medium. The final working solution containing 0.1% DMSO had no influence on cell viability.

### 4.3. Cell Viability Assay

The cytotoxic effect of rhein on HepaRG cells was assessed using an MTT assay. Cells were plated into 96-well plates at a density of 5.0 × 10^3^ cells/well. The next day, the cells were treated with 0, 50, 75, and 100 μM rhein for 24 and 48 h. DMSO (0.1%) was used as the untreated control. Then, 100 μL MTT working solution (0.5 mg/mL) was added. After incubating for 2–4 h at 37 °C, the culture supernatants were removed from the wells and the purple formazan crystals were dissolved in 150 μL DMSO. Lastly, a microplate reader (Thermo, Multiskan GO, Waltham, MA, USA) was used to measure the absorbance of the formazan solution at 570 nm. The IC_50_ values were calculated by a probit model.

For the measurement of lactate dehydrogenase (LDH), the cells were seeded into 96-well plates overnight and then treated with various concentrations of rhein for 24 h. The culture supernatant was harvested and LDH activity was measured with a commercial kit following the manufacturer’s instructions. All of the experimental results were performed three times.

### 4.4. Morphological Apoptosis

The morphological changes of the nuclei could be clearly observed by the staining of DNA with the fluorescent dye DAPI [47]. HepaRG cells at a density of 4.0 × 10^5^ cells/well were plated in 6-well plates and treated with 0, 50, 75, and 100 μM rhein for 24 h. The cells were collected and washed once with PBS and fixed with 4% paraformaldehyde for 20 min at room temperature. The cells were then stained and incubated in the dark with a DAPI solution for 10 min at room temperature. After being washed twice with PBS, the apoptotic cells were examined and photographed using an inverted Olympus IX71 fluorescence microscope (Tokyo, Japan) at 200×.

### 4.5. Apoptosis Analysis

Apoptosis was detected using an Annexin V-FITC Detection Kit (Nanjing, China) and determined by flow cytometry [48]. Cells were plated in a 6-well plate (4.0 × 10^5^ cells/well) and incubated with rhein at doses ranging from 0 μM to 100 μM for 24 h at 37 °C. The cells were collected and washed with PBS. The cells were re-suspended in 295 μL binding buffer and incubated with 5 µL Annexin V-fluoresce isothiocyanate (FITC) and 10 µL propidium iodide (PI) at room temperature in the dark. Then, the cells were washed and re-suspended with PBS. All of the samples were immediately analyzed by a flow cytometer (BD FACSCanto II, Franklin Lakes, NJ, USA). The total percentage of apoptotic cells is expressed as the summation of both early and late apoptotic subpopulations (Annexin V-FITC positive).

### 4.6. Measurement of Intracellular ROS and GSH

Generation of intracellular ROS was determined using the 2,7-dichlorofluorescin diacetate (DCFH-DA) fluorescent dye [49,50]. DCFH-DA probe is a non-polar compound that lightly diffuses into cells and is then hydrolyzed by intracellular esterase to generate DCFH, which is captured in the cells. Therefore, intracellular ROS oxidized DCFH to form the highly fluorescent compound 2,7-dichlorofluorescein (DCF), which is measured by flow cytometry. In this assay, cells were seeded in 6-well plates at a density of 4 × 10^5^ cells/well exposed to rhein (0–100 μM) for 24 h. The cells were incubated with 10 μM DCFH-DA for 30 min at 37 °C in the dark. Subsequently, the cells were harvested, washed twice with PBS, and re-suspended for analysis. The fluorescence was detected using a flow cytometer (BD FACS Canto II, New Jersey, USA).

For the measurement of GSH, cells were exposed to different concentrations of rhein or the control group for 24 h, lysed, and homogenized. The cell supernatant was harvested and assessed for GSH using a GSH assay kit (Jiancheng, Nanjing, China) according to the manufacturer’s instructions.

### 4.7. Measurement of Mitochondrial Membrane Potential

The mitochondrial membrane potential (MMP) was evaluated using a mitochondria-specific lipophilic cationic fluorescence dye JC-1 (Beyotime, Nanjing, China), which is able to selectively enter the mitochondria [51]. HepaRG cells were cultured in 6-well plates and incubated with different concentrations of rhein for 24 h. Afterward, the cells were collected and stained with JC-1 working solution (10 μM) for 30 min at 37 °C in the dark, washed twice, and re-suspended with PBS. The changes in MMP were measured and analyzed by a flow cytometer.

### 4.8. Cell Cycle Analysis

The effects of rhein on cell cycle distribution were analyzed by flow cytometry [52]. In this assay, HepaRG cells (4.0 × 10^5^ cells/well) were seeded in 6-well plates and treated with rhein (0–100 µM) for 24 h. The cells were subsequently harvested and fixed with 70% ice-cold ethanol at 4 °C overnight. The cells were then centrifuged (1000 rpm for 5 min) and incubated with PI/RNase (Beyotime, Nanjing, China) staining buffer for 30 min at 37 °C in the absence of light. Ultimately, all the samples were passed through a nylon mesh filter (Jian BO, Shuyang, China) and detected by flow cytometry. In addition, this type of analysis has been used to detect apoptosis-related DNA fragmentation patterns at the single cell level, which is evidenced by an increase in the sub-G0/G1 cell subpopulation [53].

### 4.9. Western Blot Analysis

HepaRG cells were seeded in 6-well plates and incubated with various concentrations of rhein. Then, the cells were collected and lysed with ice-cold RIPA buffer for 30 min. Subsequently, the lysates were centrifuged for 10 min at 12,000 rpm. A bicinchoninic acid assay (BCA) protein assay kit (Dinguo Changsheng Biotechnology, Beijing, China) was used to determine total protein concentration of the supernatant. In a parallel experiment, the mitochondrial and cytosolic fractions were separated using the ProteoExtract^®^ Cytosol/Mitochondria Fractionation Kit (Millipore, Billerica, MA, USA) according to the manufacturer’s instructions. Sodium dodecyl sulfate-polyacrylamide gel electrophoresis (SDS-PAGE) was done by loading equal amounts of target protein per lane and then transferring to a polyvinylidene fluoride (PVDF) membrane (Pall, New York, USA). The membranes were blocked with 5% skim milk in TBST (25 mM Tris, 150 mM NaCl, 0.1% Tween 20, pH 7.4) buffer for 1 h and then incubated with primary antibodies overnight at 4 °C [54]. After being washed four times with TBST, the membranes were further incubated with corresponding secondary antibodies at room temperature for 1 h. The target proteins were visualized with an ECL Western blotting detection reagent (Pierce, Appleton, WI, USA). All the experimental results were repeated at least three times.

### 4.10. Statistical Analysis

Each experimental result was repeated in triplicate and data are expressed as the mean ± SD. Data were processed using 17.0 SPSS software (SPSS Inc., Chicago, USA). Statistical significance was analyzed using one-way ANOVA analysis and LSD test. * *p* < 0.05 was considered to be statistically significant.

## References

[1] Yuan Y., Zheng J., Wang M., Li Y., Ruan J., Zhang H. (2016). Metabolic Activation of Rhein: Insights into the Potential Toxicity Induced by Rhein-Containing Herbs. J. Agric. Food. Chem..

[2] Lin S., Fujii M., Hou D.X. (2003). Rhein induces apoptosis in HL-60 cells via reactive oxygen species-independent mitochondrial death pathway. Arch. Biochem. Biophys..

[3] Shi P., Huang Z., Chen G. (2008). Rhein induces apoptosis and cell cycle arrest in human hepatocellular carcinoma BEL-7402 cells. Am. J. Chin. Med..

[4] Chang S.J., Huang S.H., Lin Y.J., Tsou Y.Y., Lin C.W. (2014). Antiviral activity of Rheum palmatum methanol extract and chrysophanol against Japanese encephalitis virus. Arch. Pharm. Res..

[5] Gao Y., Chen X., Fang L., Liu F., Cai R., Peng C., Qi Y. (2014). Rhein exerts pro- and anti-inflammatory actions by targeting IKKβ inhibition in LPS-activated macrophages. Free Radic. Biol. Med..

[6] Smolarz H.D., Swatko-Ossor M., Ginalska G., Medyńska E. (2013). Antimycobacterial effect of extract and its components from Rheum rhaponticum. J. AOAC Int..

[7] Chen Y.Y., Chiang S.Y., Lin J.G., Yang J.S., Ma Y.S., Liao C.L., Lai T.Y., Tang N.Y., Chung J.G. (2010). Emodin, aloe-emodin and rhein induced DNA damage and inhibited DNA repair gene expression in SCC-4 human tongue cancer cells. Anticancer Res..

[8] Lin M.L., Chung J.G., Lu Y.C., Yang C.Y., Chen S.S. (2009). Rhein inhibits invasion and migration of human nasopharyngeal carcinoma cells in vitro by down-regulation of matrix metalloproteinases-9 and vascular endothelial growth factor. Oral Oncol..

[9] Hsia T.C., Yang J.S., Chen G.W., Chiu T.H., Lu H.F., Yang M.D., Yu F.S., Liu K.C., Lai K.C., Lin C.C. (2009). The roles of endoplasmic reticulum stress and Ca^2+^ on rhein-induced apoptosis in A-549 human lung cancer cells. Anticancer Res..

[10] Li Y., Xu Y., Lei B., Wang W., Ge X., Li J. (2012). Rhein induces apoptosis of human gastric cancer SGC-7901 cells via an intrinsic mitochondrial pathway. Braz. J. Med. Biol. Res..

[11] Lin M.L., Chen S.S., Lu Y.C., Liang R.Y., Ho Y.T., Yang C.Y., Chung J.G. (2007). Rhein induces apoptosis through induction of endoplasmic reticulum stress and Ca^2+^-dependent mitochondrial death pathway in human nasopharyngeal carcinoma cells. Anticancer Res..

[12] Lian Y., Xie L., Chen M., Chen L. (2014). Effects of an astragalus polysaccharide and rhein combination on apoptosis in rats with chronic renal failure. Evid. Based Complement. Altern. Med..

[13] Zhao X., Li J., Zhu S., Liu Y., Zhao J., Wan M., Tang W. (2014). Rhein Induces a Necrosis-Apoptosis Switch in Pancreatic Acinar Cells. Evid. Based Complement. Altern. Med..

[14] KoraMagazi A., Wang D., Yousef B., Guerram M., Yu F. (2016). Rhein triggers apoptosis via induction of endoplasmic reticulum stress, caspase-4 and intracellular calcium in primary human hepatic HL-7702 cells. Biochem. Biophys. Res. Commun..

[15] Kuo P.L., Hsu Y.L., Ng L.T., Lin C.C. (2004). Rhein inhibits the growth and induces the apoptosis of Hep G2 cells. Planta Med..

[16] Panigrahi G.K., Ch R., Mudiam M.K., Vashishtha V.M., Raisuddin S., Das M. (2015). Activity-Guided Chemo Toxic Profiling of Cassia occidentalis (CO) Seeds: Detection of Toxic Compounds in Body Fluids of CO-Exposed Patients and Experimental Rats. Chem. Res. Toxicol..

[17] Panigrahi G.K., Yadav A., Srivastava A., Tripathi A., Raisuddin S., Das M. (2015). Mechanism of Rhein-Induced Apoptosis in Rat Primary Hepatocytes: Beneficial Effect of Cyclosporine A. Chem. Res. Toxicol..

[18] Dong Z., Lei Q., Yang R., Zhu S., Ke X.X., Yang L., Cui H., Yi L. (2017). Inhibition of neurotensin receptor 1 induces intrinsic apoptosis via let-7a-3p/Bcl-w axis in glioblastoma. Br. J. Cancer.

[19] AnvariFar H., Amirkolaie A.K., Miandare H.K., Ouraji H., Jalali M.A., Üçüncü S.İ. (2016). Apoptosis in fish: Environmental factors and programmed cell death. Cell Tissue Res..

[20] Zhou B., Wang H., Xue F., Wang X., Fei C., Wang M., Zhang T., Yao X., He P. (2010). Effects of diclazuril on apoptosis and mitochondrial transmembrane potential in second-generation merozoites of Eimeria tenella. Vet. Parasitol..

[21] Julien O., Wells J.A. (2017). Caspases and their substrates. Cell Death Differ..

[22] Cui Y., Lu P., Song G., Liu Q., Zhu D., Liu X. (2016). Involvement of PI3K/Akt, ERK and p38 signaling pathways in emodin-mediated extrinsic and intrinsic human hepatoblastoma cell apoptosis. Food Chem. Toxicol..

[23] Chen T.C., Lai K.C., Yang J.S., Liao C.L., Hsia T.C., Chen G.W., Lin J.J., Lin H.J., Chiu T.H., Tang Y.J. (2009). Involvement of reactive oxygen species and caspase-dependent pathway in berberine-induced cell cycle arrest and apoptosis in C6 rat glioma cells. Int. J. Oncol..

[24] Jian K.L., Zhang C., Shang Z.C., Yang L., Kong L.Y. (2017). Eucalrobusone C suppresses cell proliferation and induces ROS-dependent mitochondrial apoptosis via the p38 MAPK pathway in hepatocellular carcinoma cells. Phytomedicine.

[25] Alabsi A.M., Lim K.L., Paterson I.C., Ali-Saeed R., Muharram B.A. (2016). Cell Cycle Arrest and Apoptosis Induction via Modulation of Mitochondrial Integrity by Bcl-2 Family Members and Caspase Dependence in Dracaena cinnabari-Treated H400 Human Oral Squamous Cell Carcinoma. Biomed Res. Int..

[26] Smith M.C., Hymery N., Troadec S., Pawtowski A., Coton E., Madec S. (2017). Hepatotoxicity of fusariotoxins, alone and in combination, towards the HepaRG human hepatocyte cell line. Food Chem. Toxicol..

[27] Wu Y., Geng X.C., Wang J.F., Miao Y.F., Lu Y.L., Li B. (2016). The HepaRG cell line, a superior in vitro model to L-02, HepG2 and hiHeps cell lines for assessing drug-induced liver injury. Cell Biol. Toxicol..

[28] Fleury C., Mignotte B., Vayssière J.L. (2002). Mitochondrial reactive oxygen species in cell death signaling. Biochimie.

[29] Sauer H., Wartenberg M., Hescheler J. (2001). Reactive Oxygen Species as Intracellular Messengers during Cell Growth and Differentiation. Cell Physiol. Biochem..

[30] Garcia-Ruiz C., Fernández-Checa J.C. (2010). Redox regulation of hepatocyte apoptosis. J. Gastroenterol. Hepatol..

[31] Wu Y., Shamoto-Nagai M., Maruyama W., Osawa T., Naoi M. (2017). Phytochemicals prevent mitochondrial membrane permeabilization and protect SH-SY5Y cells against apoptosis induced by PK11195, a ligand for outer membrane translocator protein. J. Neural Transm..

[32] Yang C.L., Ma Y.G., Xue Y.X., Liu Y.Y., Xie H., Qiu G.R. (2012). Curcumin induces small cell lung cancer NCI-H446 cell apoptosis via the reactive oxygen species-mediated mitochondrial pathway and not the cell death receptor pathway. DNA Cell Biol..

[33] Wang J.B., Ma Y.G., Xue Y.X., Liu Y.Y., Xie H., Qiu G.R. (2009). Effect of processing on the chemical contents and hepatic and renal toxicity of rhubarb studied by canonical correlation analysis. Yao Xue Xue Bao.

[34] Boonstra J., Post J.A. (2004). Molecular events associated with reactive oxygen species and cell cycle progression in mammalian cells. Gene.

[35] Song Y., Li X., Li Y., Li N., Shi X., Ding H., Zhang Y., Li X., Liu G., Wang Z. (2014). Non-esterified fatty acids activate the ROS-p38-p53/Nrf2 signaling pathway to induce bovine hepatocyte apoptosis in vitro. Apoptosis.

[36] Kannan K., Jain S.K. (2000). Oxidative stress and apoptosis. Pathophysiology.

[37] Chung Y.M., Bae Y.S., Lee S.Y. (2003). Molecular ordering of ROS production, mitochondrial changes, and caspase activation during sodium salicylate-induced apoptosis. Free Radic. Biol. Med..

[38] Bennett M.R. (2001). Reactive oxygen species and death: Oxidative DNA damage in atherosclerosis. Circ. Res..

[39] Vousden K.H., Lu X. (2002). Live or let die: The cell’s response to p53. Nat. Rev. Cancer..

[40] Fridman J.S., Lowe S.W. (2003). Control of apoptosis by p53. Oncogene.

[41] Lai W.W., Yang J.S., Lai K.C., Kuo C.L., Hsu C.K., Wang C.K., Chang C.Y., Lin J.J., Tang N.Y., Chen P.Y. (2009). Rhein induced apoptosis through the endoplasmic reticulum stress, caspase- and mitochondria-dependent pathways in SCC-4 human tongue squamous cancer cells. In Vivo.

[42] Cullen S.P., Brunet M., Martin S.J. (2010). Granzymes in cancer and immunity. Cell Death Differ..

[43] Schleich K., Lavrik I.N. (2013). Mathematical modeling of apoptosis. Cell Commun. Signal..

[44] Ip S.W., Weng Y.S., Lin S.Y., Mei D.Y., Tang N.Y., Su C.C., Chung J.G. (2007). The role of Ca^+2^ on rhein-induced apoptosis in human cervical cancer Ca Ski cells. Anticancer Res..

[45] Heo S.K., Yun H.J., Park W.H., Park S.D. (2009). Rhein Inhibits TNF-α-Induced Human Aortic Smooth Muscle Cell Proliferation via Mitochondrial-Dependent Apoptosis. J. Vasc. Res..

[46] Wen X., Lin Z.Q., Liu B., Wei Y.Q. (2012). Caspase-mediated programmed cell death pathways as potential therapeutic targets in cancer. Cell Prolif..

[47] Kuo H.M., Tsai H.C., Lin Y.L., Yang J.S., Huang A.C., Yang M.D., Hsu S.C., Chung M.C., Gibson W.W., Chung J.G. (2009). Mitochondrial-dependent caspase activation pathway is involved in baicalein-induced apoptosis in human hepatoma J5 cells. Int. J. Oncol..

[48] Chan S.F., Chen Y.Y., Lin J.J., Liao C.L., Ko Y.C., Tang N.Y., Kuo C.L., Liu K.C., Chung J.G. (2017). Triptolide induced cell death through apoptosis and autophagy in murine leukemia WEHI-3 cells in vitro and promoting immune responses in WEHI-3 generated leukemia mice in vivo. Environ. Toxicol..

[49] Du X., Shi Z., Peng Z., Zhao C., Zhang Y., Wang Z., Li X., Liu G., Li X. (2017). Acetoacetate Induces Hepatocytes Apoptosis by the ROS-Mediated MAPKs Pathway in Ketotic Cows. J. Cell. Physiol..

[50] Afri M., Frimer A.A., Cohen Y. (2004). Active oxygen chemistry within the liposomal bilayer. Part IV: Locating 2′,7′-dichlorofluorescein (DCF), 2′,7′-dichlorodihydrofluorescein (DCFH) and 2′,7′-dichlorodihydrofluorescein diacetate (DCFH-DA) in the lipid bilayer. Chem. Phys. Lipids.

[51] He Z., Pu L., Yuan C., Jia M., Wang J. (2017). Nutrition deficiency promotes apoptosis of cartilage endplate stem cells in a caspase-independent manner partially through upregulating BNIP3. Acta Biochim. Biophys. Sin..

[52] Song X.L., Zhang Y.J., Wang X.F., Zhang W.J., Wang Z., Zhang F., Zhang Y.J., Lu J.H., Mei J.W., Hu Y.P. (2017). Casticin induces apoptosis and G0/G1 cell cycle arrest in gallbladder cancer cells. Cancer Cell Int..

[53] Robles-Escajeda E., Lerma D., Nyakeriga A.M., Ross J.A., Kirken R.A., Aguilera R.J., Varela-Ramirez A. (2013). Searching in mother nature for anti-cancer activity: Anti-proliferative and pro-apoptotic effect elicited by green barley on leukemia/lymphoma cells. PLoS ONE.

[54] Lai S.H., Jiang G.B., Yao J.H., Li W., Han B.J., Zhang C., Zeng C.C., Liu Y.J. (2015). Cytotoxic activity, DNA damage, cellular uptake, apoptosis and western blot analysis of ruthenium(II) polypyridyl complex against human lung decarcinoma A549 cell. J. Inorg. Biochem..

